# Predicting central lymph node metastasis in patients with papillary thyroid carcinoma based on ultrasound radiomic and morphological features analysis

**DOI:** 10.1186/s12880-023-01085-4

**Published:** 2023-08-24

**Authors:** Xiang Yan, Xurong Mou, Yanan Yang, Jing Ren, Xingxu Zhou, Yifei Huang, Hongmei Yuan

**Affiliations:** Sichuan Key Laboratory of Medical Imaging, Department of Ultrasound, Affiliated Hospital of North Sichuan Medical College, Nanchong, 637000 China

**Keywords:** Papillary thyroid carcinoma, Ultrasound, Radiomics, Lymph node metastasis

## Abstract

**Objectives:**

To build a combined model based on the ultrasound radiomic and morphological features, and evaluate its diagnostic performance for preoperative prediction of central lymph node metastasis (CLNM) in patients with papillary thyroid carcinoma (PTC).

**Method:**

A total of 295 eligible patients, who underwent preoperative ultrasound scan and were pathologically diagnosed with unifocal PTC were included at our hospital from October 2019 to July 2022. According to ultrasound scanners, patients were divided into the training set (115 with CLNM; 97 without CLNM) and validation set (45 with CLNM; 38 without CLNM). Ultrasound radiomic, morphological, and combined models were constructed using multivariate logistic regression. The diagnostic performance was assessed by the area under the curve (AUC) of the receiver operating characteristic curve, accuracy, sensitivity, and specificity.

**Results:**

A combined model was built based on the morphology, boundary, length diameter, and radiomic score. The AUC was 0.960 (95% CI, 0.924–0.982) and 0.966 (95% CI, 0.901–0.993) in the training and validation set, respectively. Calibration curves showed good consistency between prediction and observation, and DCA demonstrated the clinical benefit of the combined model.

**Conclusion:**

Based on ultrasound radiomic and morphological features, the combined model showed a good performance in predicting CLNM of patients with PTC preoperatively.

**Supplementary Information:**

The online version contains supplementary material available at 10.1186/s12880-023-01085-4.

## Introduction

The incidence of thyroid cancer has increased dramatically worldwide in recent years [1]. Papillary thyroid carcinoma (PTC), accounting for 80–89% of thyroid carcinoma, is the most common thyroid carcinoma [2, 3]. Cervical lymph node metastasis, which occurs in approximately 35–80% of patients with PTC, is the most important risk factor related to recurrence and poor overall survival [4, 5]. As central lymph node metastasis (CLNM) is the first station of metastasis and the most frequently involved area, central compartment neck dissection (CCND) is often performed for patients with positive CLNM, which can significantly reduce the recurrence rate and mortality [6]. However, approximately 38% of PTC patients have been reported to have CLNM even in patients with no clinical evidence of nodal metastasis [7]. As preventive CCND increases the incidence of surgical complications, such as parathyroid and laryngeal recurrent nerve injuries, preventive CCND is controversial in CLNM-negative patients [8]. Therefore, to reduce the unnecessary CCND, an accurate and non-invasive method for preoperative assessment of CLNM is essential for selecting the optimal treatment strategy for patients with PTC.

Ultrasonography (US) is the preferred modality for the preoperative evaluation of patients with PTC and it is widely used to screen thyroid nodules and lymph node metastases. Studies have demonstrated that several ultrasound morphological features are associated with CLNM [9–11], such as tumor size, extrathyroidal extension, microcalcification, etc. However, the diagnostic performance show greatly varying degrees among different US physicians due to the subjectivity of the experience and application of diagnostic criteria [12–15]. Therefore, an effective and stable method is needed to evaluate the central lymph nodes of patients with PTC. Radiomics is an emerging technique, which can not only improve the accuracy and consistency of disease diagnosis but also reduce the time to output results. Based on these superiorities, several limited studies have investigated radiomics to predict the CLNM in PTC patients [16, 17]. However, the models in these studies are based on US-reported central lymph nodes status. As the US has a low detection rate for central lymph nodes owing to the influence of air, bone, or glandular tissues [18], it may lead to some impact on the accuracy of the model. Thus, the radiomic model in this study is based on the thyroid nodules themselves, which can avoid the above problems to some degree. In addition, different from previous studies that only determine the risk factors of CLNM [19, 20], the aim of our study is not only to identify risk factors for predicting CLNM, but also to develop and validate the models for predicting CLNM. Moreover, few studies have evaluated the overall diagnostic performance of the combined model, which has integrated the radiomics with conventional morphological features.

Therefore, to fill the gap of the available literature, our study aims to build an ultrasound radiomic model and compare its diagnostic value with a conventional morphological model for predicting CLNM in patients with PTC. We further construct a combined model based on ultrasound radiomic and morphological features, to assist clinical selection of the best treatment.

## Materials and methods

### Patients

**Fig. 1:**
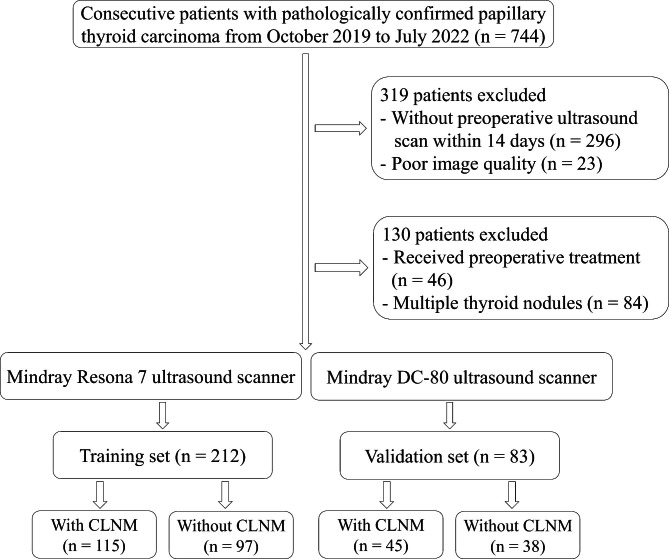
The flow diagram of patients selection. CLNM, central lymph node metastasis

The retrospective study was approved by our institutional review board, and the informed consent was waived. Consecutively patients with pathologically confirmed PTC from October 2019 to July 2022 were retrospectively collected in our hospital. Inclusion criteria were as follows: (1) patients who underwent thyroidectomy and lymph node dissection in the central region of the neck; and (2) patients who underwent ultrasound examination within two weeks before surgery. Exclusion criteria were as follows: (1) patients who had received preoperative treatment (radiofrequency or microwave ablation, radiotherapy, radioiodine therapy, or chemotherapy); (2) multiple thyroid nodules with confirmed PTC in one lobe; and (3) poor image quality. Details of the patient recruitment in this study were shown in the flow chart (Fig. 1).

Finally, a total of 295 patients with PTC were included in this study, including 49 men and 246 women, with a mean age of 43.23 (20–73) years.

### Ultrasound radiomic model

#### Image acquisition

To preserve maximum longitudinal images of bilateral lobe nodules of the thyroid gland according to thyroid and neck ultrasound examination specifications, images obtained with Mindray Resona 7 and Mindray DC-80 color Doppler ultrasound diagnostic instruments from Mindray, L14–5WU, and L14–5WE line array probes with frequencies from 4 to 14 MHz. were included. According to the different ultrasound scanners, patients who underwent Mindray Resona 7 scan were included in the training set (115 with CLNM, 97 without CLNM), while those who underwent the Mindray DC-80 scan were enrolled in the validation set (45 with CLNM, 38 without CLNM).

#### Image segmentation

**Fig. 2:**
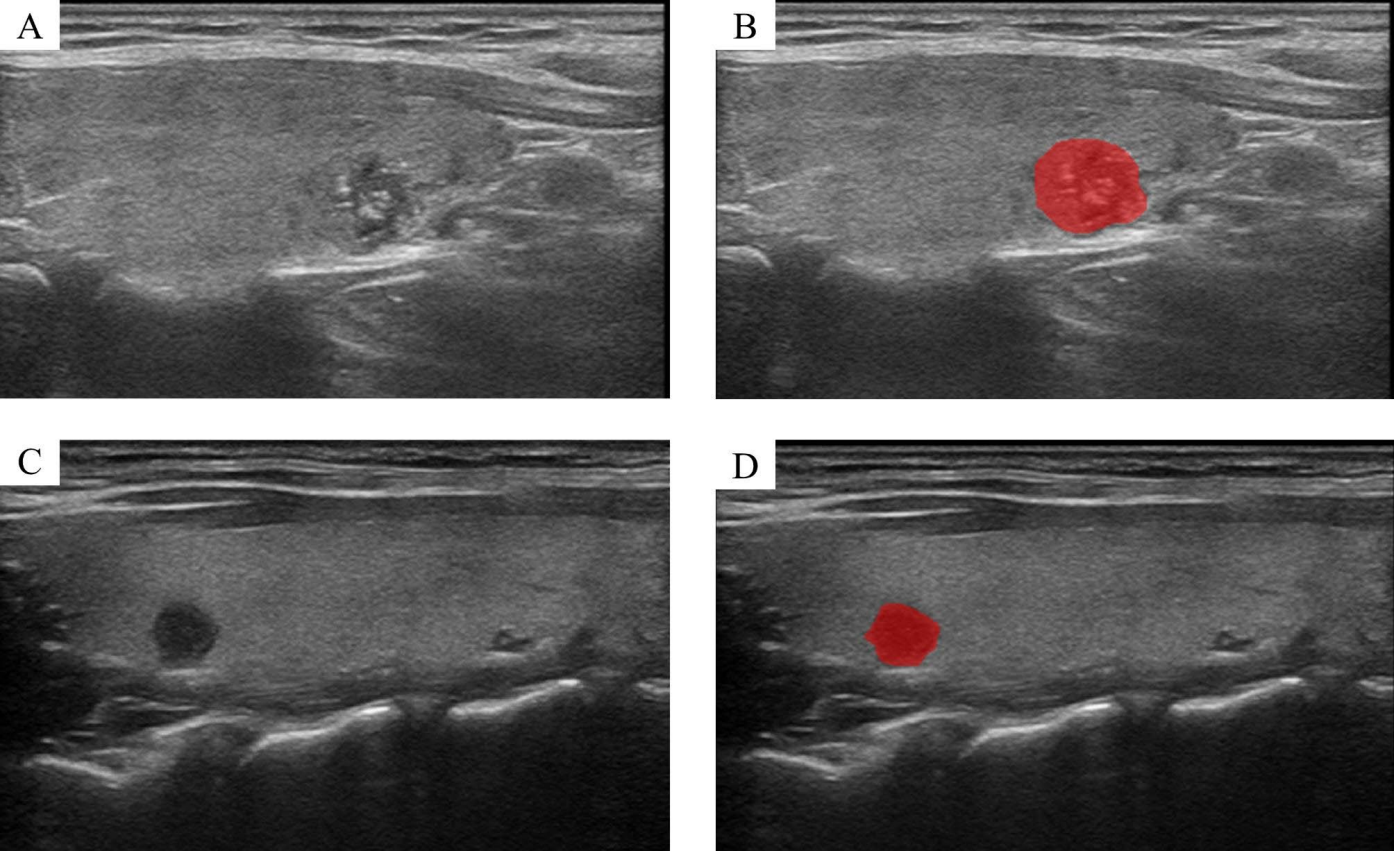
Representative images and segmentation results of thyroid nodules. (**A, B**) A female with central lymph node metastasis. (**C, D**) A female without central lymph node metastasis. (**A, C**) Original images and (**B, D**) segmentation results

The two-dimensional (2D) ultrasound images included in this study were imported into the ITK-SNAP software in DICOM format, and the region of interest (ROI) of the lesion was manually contoured by a US physician with 5 years of experience in thyroid ultrasound diagnosis. To evaluate the inter-observer agreement, another US physician with 10 years of experience in thyroid ultrasound diagnosis outlined 30 randomly selected cases. Both US physicians were blind to the pathological result. The reliability was evaluated through the intraclass correlation coefficient (ICC). The features with ICCs > 0.75 indicated a high reproducibility, which was reserved for further analysis. The representative images were shown in Fig. 2.

#### Feature extraction

Owing to the different acquisition parameters of the two ultrasound scanners, all the original images were first resampled at a spatial resolution of 1 × 1 × 1 mm^3^ through a linear interpolation algorithm to ensure the rotationally invariant and comparable texture features between different scanners [21]. In addition, a fixed bin count of 32 was used to discretize voxel intensity values and reduce the noise. Then, the PyRadiomics package (https://pyradiomics.readthedocs.io) was used to extract the radiomic features [22], including first-order, morphological, and texture features [gray-level cooccurrence matrix (GLCM), gray-level dependence matrix (GLDM), gray-level run length matrix (GLRLM), gray-level size zone matrix (GLSZM), and neighborhood gray-tone difference matrix (NGTDM)]. Gray level co-occurrence matrix (GLCM) and gray-level dependence matrix (GLDM) features are computed from each 3D directional matrix and averaged over the 3D directions, and gray level size zone matrix (GLSZM), neighboring grey level dependence matrix (NGLDM), and neighboring gray-tone difference matrix (NGTDM) features are computed from a 3D matrix. Details of radiomic features were available at https://pyradiomics.readthedocs.io/en/latest/features.html.Then, to obtain the filtered derived image and extract the corresponding ultrasound radiomic features, the wavelet transform is applied to the original image. Finally, a total of 464 radiomic features were extracted.

#### Model construction

Z-score normalization was first applied to standardize the radiomic features in the training set. The least absolute shrinkage and selection operator (LASSO) was used to avoid overfitting the radiomic features and select the most significant predictive features. To find an optimal regulation weight in LASSO logistic regression, 10-fold cross-validation with minimum criteria was used, in which the value of λ yielded the minimum binomial deviance. Then, multivariate logistic regression analysis, with stepwise regression selection method and Akaike information criterion (AIC) was used to construct the final ultrasound radiomic model. Ultimately, we validate the model with an independent validation set. The selected features were linearly weighted to calculate the radiomic score (Rad-score).

### Ultrasound morphological model

Ultrasound morphological features in this study included: location, length diameter, morphology, boundary, color doppler flow imaging, and microcalcifications of the nodules. Two ultrasonographers with 5 and 10 years of experience in diagnostic thyroid ultrasound completed the thyroid nodule morphologic feature evaluation independently. Any disagreement was resolved by negotiation and discussion. If disagreement was insisted, a senior ultrasonographer with 15 years of experience made the final determination. All ultrasonographers were unaware of the corresponding pathologic histologic findings before evaluating the images. All morphological features were included in a univariate logistic regression analysis to assess the predictive power of central lymph node metastasis. Subsequently, features with *P* < 0.05 were placed into a multivariate logistic regression analysis, and the final morphological model was constructed using the stepwise regression selection method and AIC.

### Building combined model

The ultrasound radiomic and morphological features with *P* < 0.05 were subjected to a multivariate logistic regression analysis, and independent risk predictors for central lymph node metastasis were determined using the stepwise regression selection method and AIC to construct the final combined model.

### Statistical analysis

As a prediction model study for diagnostic purposes, this study followed the transparent reporting of a multivariable prediction model for individual prognosis or diagnosis (TRIPOD) statement [23].

SPSS (version 25.0), R software (version 4.2.1), and MedCalc (version 19.6.4) were used for statistical analysis. Group differences were evaluated through the Mann-Whitney *U* test for continuous variables, and the chi-square test or Fisher test for categorical variables. A two-sided *P* < 0.05 was indicative of a significant difference. The diagnostic performance of the combined, ultrasound radiomic and morphological models were assessed using the area under the curve (AUC) of the receiver operating characteristic (ROC) curve. The optimal threshold value of the ROC curve was determined through the Youden index. The corresponding accuracy, sensitivity, and specificity values were calculated, and a 95% confidence interval (CI) was calculated by the Binomial exact method. The Hosmer-Lemeshow test was performed to estimate the goodness-of-fit of each model, and the calibration curves were plotted for each model, which was capable of visualizing the consistency of models. Finally, the clinical decision curve analysis (DCA) was used to evaluate the clinical application value of each model.

## Result

### Baseline characteristics

The clinical and ultrasound morphological characteristics of 295 thyroid nodules were presented in Table 1, in which there were significant differences in color doppler flow imaging, microcalcifications, morphology, boundary, and length-diameter between groups in both the training and validation sets (all *P* < 0.05). Significant differences were found in gender between groups in the training set (*P* = 0.018), but not in the test set (*P* = 0.483). No significant differences were found in age and location between groups in both the training and validation sets (all *P* > 0.05).

**Table 1:** Clinical and ultrasound morphological features of the patients

	Training set			Validation set		
	with CLNM (n = 115)	without CLNM (n = 97)	*P*	with CLNM (n = 45)	without CLNM (n = 38)	*P*
Gender			0.018^*^			0.438
Male	21	7		10	11	
Female	94	90		35	27	
Age	42.93 ± 11.95	43.43 ± 9.74	0.781	42.42 ± 12.00	44.61 ± 10.26	0.528
Microcalcification			0.001^*^			< 0.001^*^
Yes	91	57		38	18	
No	24	40		7	20	
Color doppler flow imaging			< 0.001^*^			0.014^*^
Avascularity	21	55		8	11	
Mainly peripheral vascularity	45	17		14	20	
Mainly central vascularity	37	18		19	7	
Mixed vascularity	12	7		4	0	
Location			0.510			0.143
Up	22	25		16	9	
Middle	44	34		18	12	
Down	49	38		11	17	
Morphology			< 0.001^*^			< 0.001^*^
Regular	20	57		8	22	
Irregular	95	40		37	8	
Boundary			< 0.001^*^			0.031^*^
Circumscribed	19	39		7	9	
Ill-defined	42	40		16	21	
Irregular margin	54	18		22	8	
Length diameter	1.16 ± 0.71	0.72 ± 0.45	< 0.001^*^	1.20 ± 0.75	0.63 ± 0.24	< 0.001^*^

CLNM, central lymph node metastasis. * *P* < 0.05

### Ultrasound radiomic model

**Fig. 3:**
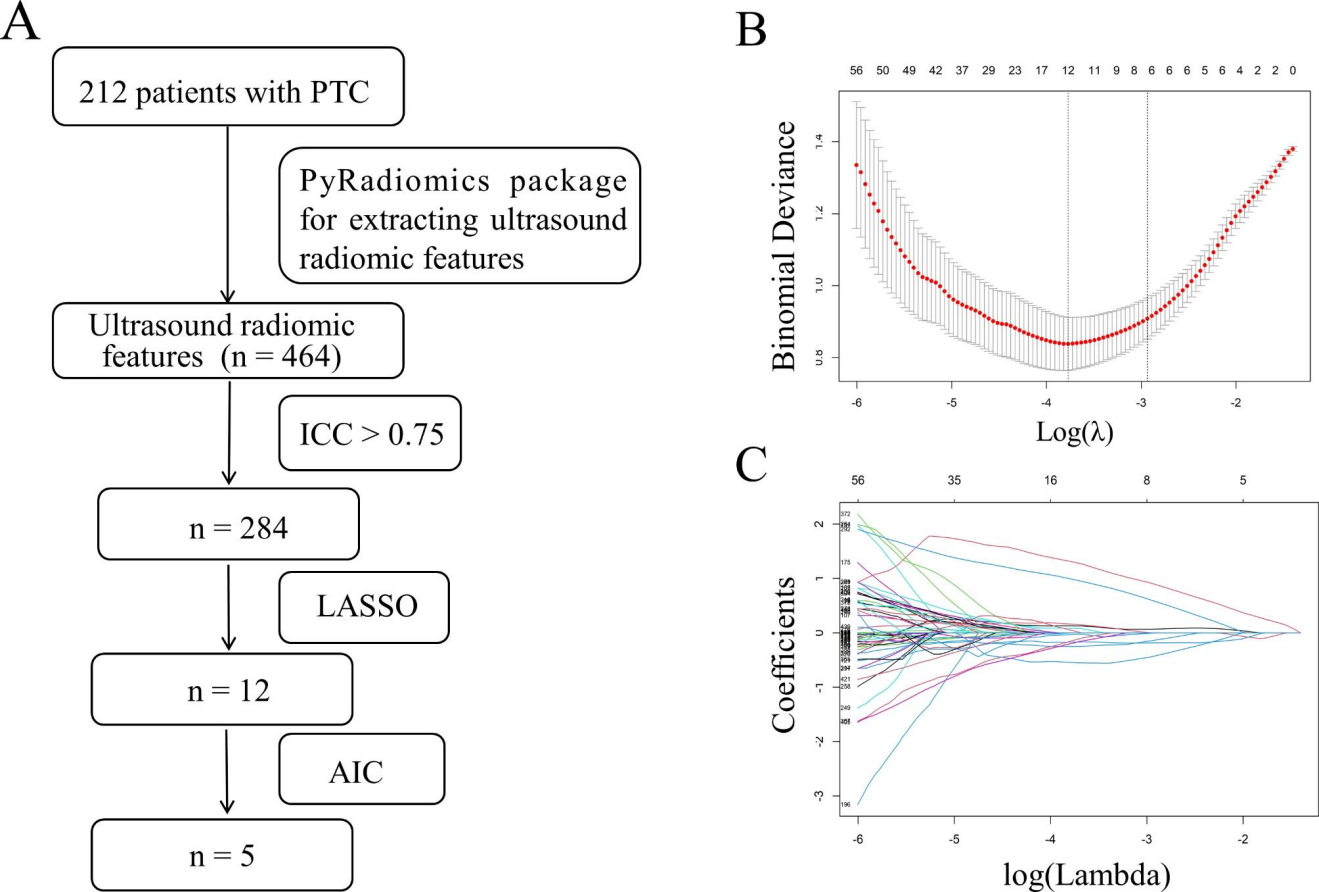
The process of the ultrasound radiomic feature selection. (**A**) Flow chart of ultrasound radiomic features selection. (**B**) Radiomic feature selection by applying the LASSO binary logistic regression model. The vertical lines on the left side of the horizontal coordinate represent the tuning parameter lambda (λ) chosen in the LASSO model using 10-fold cross-validation via minimum criteria. (**C**) Coefficient convergence plot of the screened features, with the best log (λ) value screening out 12 histological features with non-zero coefficients. LASSO, least absolute shrinkage, and selection operator; PTC, papillary thyroid carcinoma; AIC, Akaike information criterion

In total, 284 of the 464 ultrasound radiomic features had high reproducibility (ICC > 0.75). The process of the ultrasound radiomic feature selection was shown in Fig. 3. After selecting, five features remained to construct the ultrasound radiomic model. The formula was as follows: ln(P/1-P) = 0.226 + 1.457 × wavelet.HH_first_order_Median + 1.287 × wavelet.LL_first_order_Range − 1.185 × wavelet.LL_GLCM_Imc2–2.239 × wavelet.HH_GLDM_SmallDependenceHighGrayLevelEmphasis + 2.331 × wavelet.HL_GLSZM_ZoneEntropy, in which P is the probability of CLNM, with a cutoff value > 0.498. To standardize the features by Z-score before calculation, the mean and standard deviation of these features were detailed in Table 2.

**Table 2:** Means and standard deviations of ultrasound radiomic features

Features	Mean	SD
wavelet.HH_first_order_Median	− 0.035	0.145
wavelet.LL_first_order_Range	553.065	209.601
wavelet.LL_GLCM_Imc2	0.978	0.014
wavelet.HH_GLDM_SmallDependenceHighGrayLevelEmphasis	24.693	33.561
waveletHL_GLSZM_ZoneEntropy	5.199	0.178

SD, standard deviation. GLCM, gray-level cooccurrence matrix. GLDM, gray-level dependence matrix; GLSZM, gray-level size zone matrix

### Ultrasound morphological model

Univariate logistic regression analysis showed that gender, microcalcifications, color doppler flow imaging, morphology, boundary, and length-diameter were risk factors for predicting CLNM (all *P* < 0.05) (Table 3). According to the multivariate logistic regression analysis, based on the length diameter of the nodes (OR, 2.623; 95% CI, 1.199–5.738, *P* = 0.016), morphology (OR, 4.114; 95% CI, 1.896–8.926, *P* < 0.001), color doppler flow imaging (OR, 7.194; 95% CI, 2.865–18.067, *P* < 0.001) and boundary (OR, 3.558; 95% CI, 1.352–9.363, *P* = 0.016) to construct an ultrasound morphological model. The formula was: ln(P/1-P) = − 2.911 + 1.404 × morphology_(irregular)_ + 1.309 × boundary_(irregular margin)_ + 0.440 × boundary_(ill−defined)_ + 1.818 × color doppler flow imaging_(mainly central vascularity)_ + 0.933 × color doppler flow imaging_(Mainly peripheral vascularity)_ − 0.197 × color doppler flow imaging_(mixed vascularity)_ + 0.937 × length diameter, where P was the probability of CLNM, with a cutoff value > 0.747.

**Table 3:** Univariate and multivariate logistic regression analysis of ultrasound radiomic and morphological features

Characteristics	Univariate logistic regression analysis			Multivariate logistic regression analyses		
	OR	95% CI	*P*	OR	95% CI	*P*
Gender						
Male ^#^	1			/		
Female	0.348	0.141–0.859	0.022^*^	/	/	/
Age	0.996	0.972–1.021	0.739	/	/	/
Microcalcification						
No ^#^	1			/		
Yes	2.661	1.453–4.871	0.002^*^	/	/	/
Color doppler flow imaging						
Avascularity ^#^	1			/		
Mainly peripheral vascularity	5.384	2.530–11.454	< 0.001^*^	/	/	/
Mainly central vascularity	6.933	3.271–14.692	< 0.001^*^	/	/	/
Mixed vascularity	4.490	1.557–12.947	0.005^*^	/	/	/
Location						
Up ^#^	1			/		
Middle	1.471	0.711–3.043	0.298	/	/	/
Down	1.465	0.719–2.988	0.293	/	/	/
Morphology						
Regular ^#^	1			1		
Irregular	6.769	3.608–12.699	< 0.001^*^	10.466	3.016–36.323	< 0.001^*^
Boundary						
Circumscribed ^#^	1			1		
Ill-defined	2.155	1.072–4.335	0.031^*^	1.783	0.557–5.710	0.330
Irregular margin	6.158	2.865–13.233	< 0.001^*^	9.767	2.262–42.173	0.002^*^
Length diameter	4.675	2.414–9.052	< 0.001^*^	0.392	0.161–0.955	0.039^*^
Rad-score	2.718	2.065–3.579	< 0.001^*^	3.577	2.372–5.395	< 0.001^*^

OR, Odds ratio; 95% CI, 95% confidence interval; Rad-score, Radiomic score

^#^ Characteristics were set as the reference. * *P* < 0.05

**Table 4:** Diagnostic performance of each model in predicting central lymph node metastasis in the training and validation sets

		AUC (95% CI)	Accuracy	Sensitivity	Specificity
Training set	Combined model	0.956 (0.928–0.984)	0.882 (0.836–0.925)	0.922 (0.886–0.958)	0.835 (0.785–0.885)
	Ultrasound radiomic model	0.920 (0.880–0.956)	0.834 (0.790–0.889)	0.870 (0.824–0.914)	0.804 (0.751–0.858)
	Morphological model	0.836 (0.786–0.886)	0.693 (0.631–0.755)	0.522 (0.455–0.589)	0.897 (0.856–0.938)
Validation set	Combined model	0.973 (0.951–0.995)	0.916 (0.879–0.953)	0.933 (0.899–0.967)	0.895 (0.853–0.936)
	Ultrasound radiomic model	0.918 (0.836–0.967)	0.807 (0.754–0.860)	0.822 (0.771–0.874)	0.789 (0.735–0.844)
	Morphological model	0.796 (0.742–0.850)	0.614 (0.548–0.680)	0.378 (0.313–0.443)	0.895 (0.854–0.936)

95% CI, 95% confidence interval

### Combined model

According to the multivariate logistic regression analysis (Table 3), the final combined model was constructed based on the morphology (OR, 10.466; 95% CI, 3.016–36.323, *P* < 0.001), boundary (OR, 9.767; 95% CI, 2.262–42.173, *P* = 0.02), length diameter (OR, 0.392; 95% CI, 0.161–0.955, *P* = 0.039) and Rad-score (OR, 3.577; 95% CI, 2.372–5.395, *P* < 0.001) (Table 3). The formula was: ln(P/1-P) = − 1.764 + 2.348 × morphology_(irregular)_ + 2.279 × boundary_(irregular margin)_ + 0.579 × boundary_(ill−defined)_ − 0.936 × length diameter + 1.275 × Rad-score, where P is the probability of CLNM, with a cutoff value > 0.486.

### Model performance

**Fig. 4:**
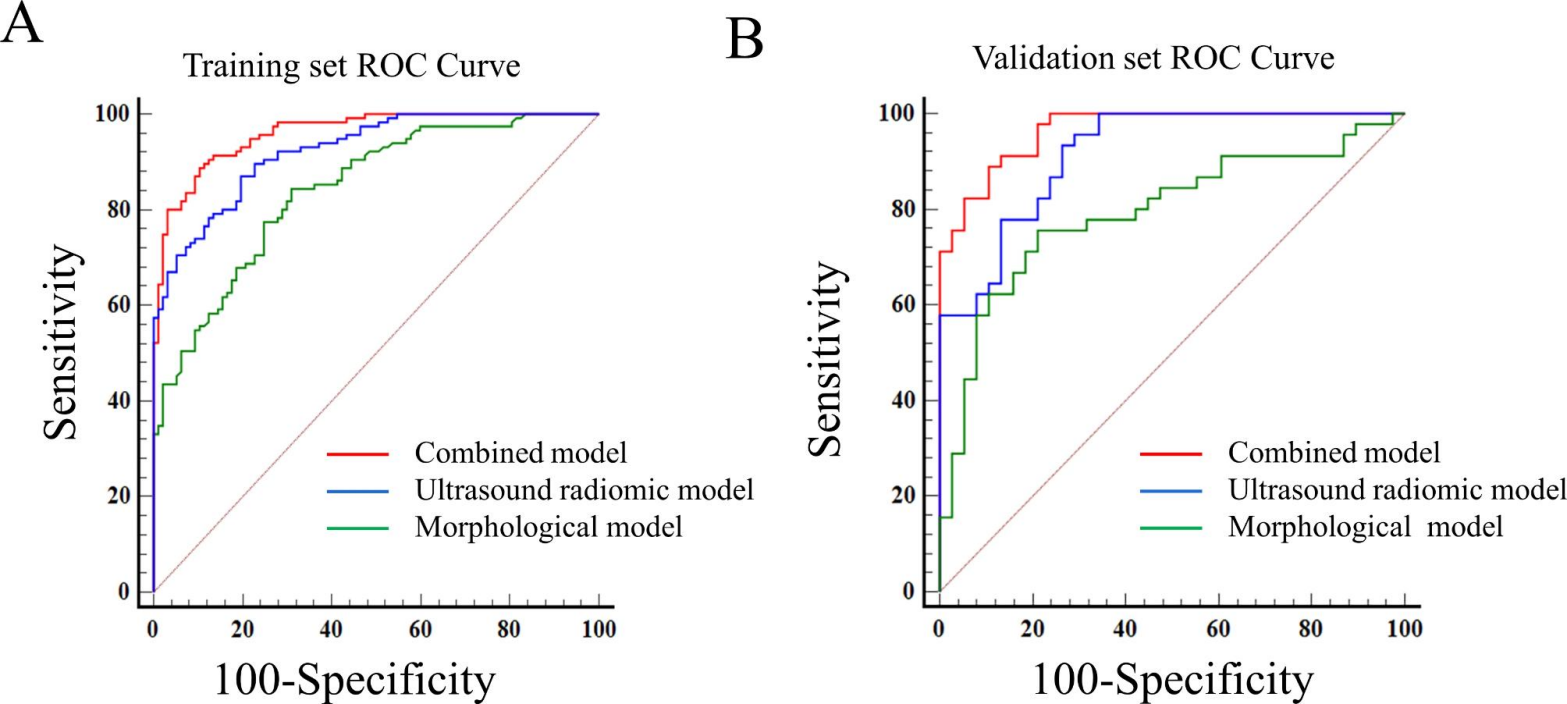
The receiver operating characteristic (ROC) curves of combined, ultrasound radiomic, and morphological models. (**A**) training set, (**B**) validation set

**Fig. 5:**
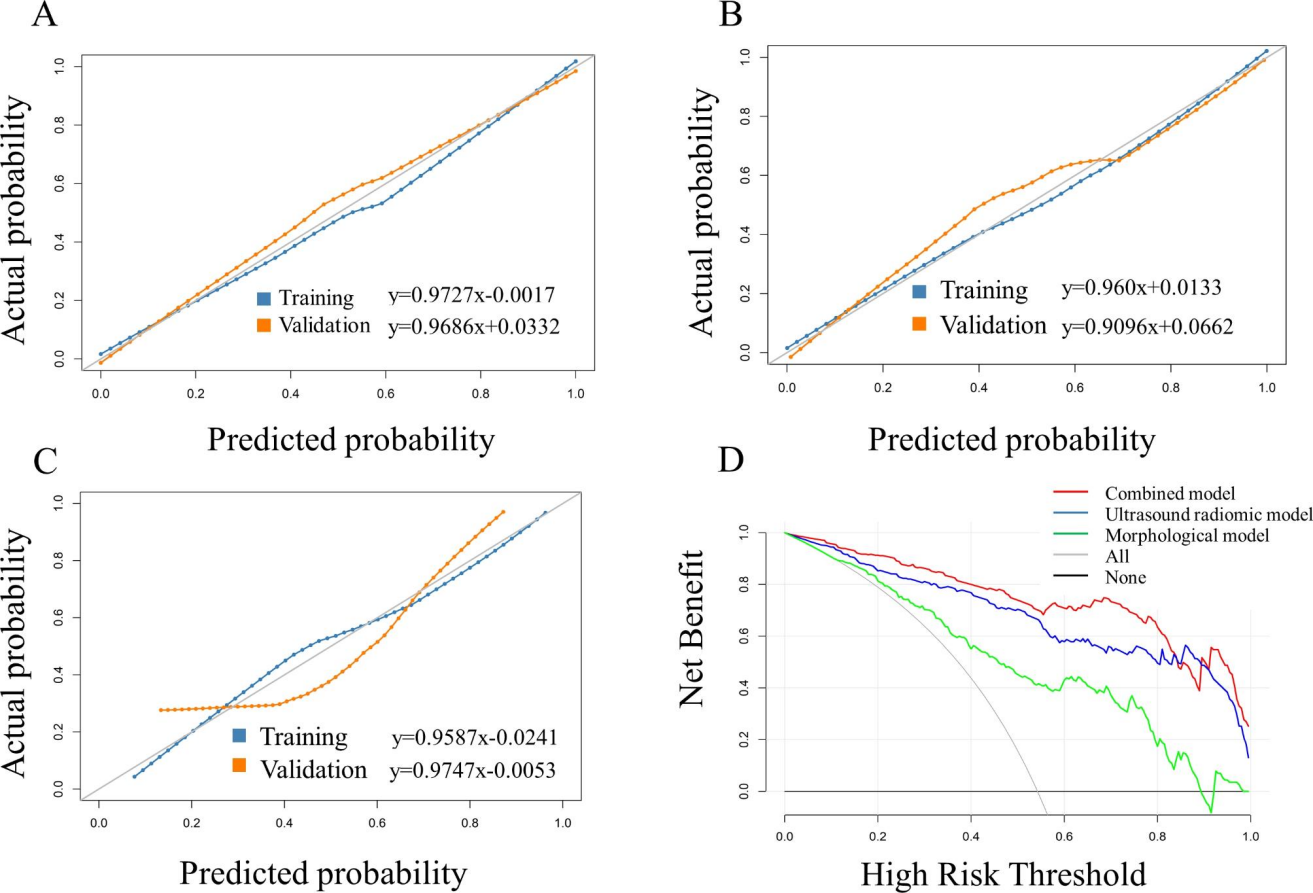
Calibration curves and clinical decision curves of each model. (**A**) Combined model calibration curve, (**B**) Ultrasound radiomic model calibration curve, (**C**) Morphological model calibration curve, (**D**) clinical decision curve

The ROC curves of the combined, ultrasound radiomic and morphological models in the training and validation sets are shown in Fig. 4. The AUCs of the models for predicting CLNM were 0.956 (95% CI, 0.928–0.984), 0.920 (95% CI, 0.875–0.953), 0.836 (95% CI, 0.786–0.886) in the training set, and 0.973 (95% CI, 0.951–0.995), 0.901 (95% CI, 0.815–0.955), 0.796 (95% CI, 0.742–0.850) in the validation set, respectively.The accuracy, sensitivity, and specificity of each model were listed in Table 4. The AUCs of the combined model were significantly higher than ultrasound radiomic and morphological models in both the training and validation sets, and the combined model showed significant higher AUC than three sonographers in the validation cohort (all *P* < 0.05) (Table 5). The Hosmer-Lemeshow test showed non-significant results for the models in the training and validation sets (all *P* > 0.05), and the calibration curves showed good consistency between prediction and observation (Fig. 5A and B C). The DCA analysis demonstrated that the combined model had a higher net clinical benefit compared with the other models (Fig. 5D).

**Table 5:** Comparison of the diagnostic performance of the models in predicting central lymph node metastasis in the training and validation sets

Model	Training set		Validation set	
	*Z*	*P*	*Z*	*P*
Combined model vs. Ultrasound radiomic model	2.864	0.004	2.019	0.044
Combined model vs. Morphological model	4.706	< 0.001	3.392	< 0.001
Ultrasound radiomic model vs. Morphological model	2.920	0.003	2.036	0.042

We concluded the TRIPOD type of this study could be type 2b.

## Discussion

PTC is the most common thyroid carcinoma with low mortality and good prognosis, with a 10-year survival rate of 97% [24]. However, the recurrence rate of PTC with cervical lymph nodes can reach 22% [25]. In particular, metastases in the central region of the neck are the most common [26]. Among the currently used imaging modalities, the efficacy of computed tomography (CT) and US in the evaluation of thyroid and CLNM are not significantly different [27], and magnetic resonance imaging (MRI) has a slightly higher diagnostic sensitivity than US [28]. However, CT is radioactive and insensitive to soft tissue; MRI is expensive and took a long time. The US is considered the imaging test of choice for the evaluation of thyroid nodules and lymph node metastases owing to its lack of radiation damage and ease of universal access. However, the US has a high specificity (93–98%) and low sensitivity (33–64%) for central lymph nodes owing to the influence of air, bone, or glandular tissues [15, 18, 27, 29]. In this study, the sensitivity of the US morphological model for the detection of CLNM was 52%, and the specificity was 90%, which is consistent with the literature. Since preoperative US is prone to miss or misdiagnosis of CLNM, prophylactic CCND can reduce the tumor recurrence rate. However, it also increases the incidence of surgical complications [30]. The Chinese and Japanese thyroid guidelines advocate aggressive prophylactic CCND, while the American Thyroid Association does not recommend prophylactic CCND [31], which is controversial. Therefore, accurate preoperative assessment of the status of central lymph node metastasis of PTC is critical for the clinical selection of whether to dissect the central lymph nodes to improve the therapeutic efficacy without increasing surgical complications.

Previous literature reported that US features of PTC, such as tumor size, microcalcifications, irregular shape and multifocality, were associated with CLNM [10, 11]. In this study, we found significant differences between the CLNM and no-CLNM groups in the morphology, boundary, length-diameter, color doppler flow imaging, and microcalcifications of the PTC lesions using univariate logistic regression. However, multivariate logistic regression showed that only the lesion length-diameter, morphology, boundary, and color doppler flow imaging were significantly associated with CLNM. Some of the US morphological features, such as microcalcifications, were different from those reported in the literature and were associated with CLNM in the univariate logistic regression analysis of this study. However, microcalcifications were not an independent risk factor for CLNM in the multivariate logistic regression analysis. This may be related to its pathological basis, such as the mummification of some benign thyroid nodules, whose US presentation is also similar to microcalcifications [32]. Ultimately, we created morphological model using the above features. However, the sensitivity of this model is only 52%. Thus, a more effective method is needed for predicting CLNM.

Radiomics is an emerging frontier discipline that can extract a large number of quantitative imaging features from medical images for deep mining of tumor biological information and analysis of tumor heterogeneity. Moreover, radiomic analysis based on image features has objectivity and is valuable in predicting clinical outcomes [33, 34]. Previously, models based on radiomic features have been applied to predict lymph node metastasis in several tumors [35, 36]. For example, shear wave elastography combined with grayscale ultrasound for predicting CLNM of PTC [37]. It shows the feasibility of applying ultrasound radiomics features to predict CLNM in patients with PTC. In this study, we used radiomic analysis to predict the presence of CLNM, and the results demonstrated that the ultrasound radiomic model could predict CLNM in PTC patients in both the training (AUC, 0.920) and validation sets (AUC, 0.901) with higher efficacy than the US morphology model, which indicates the value of radiomics in predicting the presence of CLNM in patients with PTC.

In previous studies [10], investigators have often used US morphological or radiomic features alone for the prediction of lymph node metastasis. They have not evaluated the diagnostic accuracy and additional benefit of combined US morphological and radiomic features. A much higher diagnostic value of preoperative prediction of CLNM has been reported in patients with PTC when CT and US or US and clinical are combined [27, 38]. Therefore, we developed and validated a combined model in this study for predicting CLNM in PTC patients that combine ultrasound morphological and radiomic features. The combined model had higher diagnostic efficacy and more stable sensitivity and specificity in both the training and validation sets than the morphological or radiomic models alone. The combined model provided better net clinical benefit than the ultrasound radiomic or morphological models over the most reasonable threshold probability ranges, as demonstrated by DCA. Additionally, calibration curves showed good agreement between predicted and actual values for each model. The DeLong test depicted significant differences in AUC between the ultrasound radiomic, morphological, and combined models. These results demonstrated that our combined model can greatly facilitate the preoperative individualized prediction of CLNM in patients with PTC. Many studies have been conducted on this topic. Tong et al. [17] established a nomogram based on ultrasound-reported lymph node status and radiomic features to predict CLNM in patients with PTC. However, the exploration of central lymph nodes was limited due to gas, bone, etc., which may affect the reporting of lymph node status and thus have an impact on the results, by contrast, our study was directly targeting the tumor itself, which greatly avoids the problem of displaying central lymph nodes. Zhou et al. [39] developed a radiomics nomogram based on dual-energy CT derived iodine maps, which showed favorable performance in predicting CLNM in PTC patients with an AUC of 0.837. However, CT involves radiation exposure, and iodinated contrast agents have a risk of contrast allergy. Moreover, this examination might delay radioactive iodine therapy, which limits its clinical application. Li et al. [40] develop a CT-based radiomics model to predict CLNM preoperatively in patients with PTC. Compared with the study, our combined model has better sensitivity and specificity and eliminates the need for CT examinations, which reduces the financial stress and radiation damage to patients and can be performed in the clinic to serve the patients more effectively.

With the popularity of artificial intelligence in recent years, radiomics and deep learning have been widely used in the studies of tumor imaging. For example, Abbasian Ardakani A et al. [41] used both machine learning (ML) and deep learning (DL) models on multi-center databases to predict lymph nodes metastatic preoperatively in patients with PTC. Although this was a multicenter study, its sample size was limited. This may have some implications for model construction. In addition, the model for this study was based on US images of lymph nodes. This will ignore some of the central lymph nodes that are not shown due to gas, bone, etc. This may lead to the omission of some patients with CLNM. Comparing with this study, our study is based on the tumor itself, avoiding the above makes for more accurate results. However, only internal and not external validation was performed in our study. While there is a lot of research on DL [42–44], the underlying parameters of DL algorithms were inherently in black box, there is a lack of a sufficient number of studies performing external validation on DL algorithms and a limited number of publications focusing on DL protocols, which limits the extent to which the results could be generalized. Compare with DL models, radiomics model has better protocols and fewer data requirements. This makes the model more reproducible. And the disadvantage of radiomics is that some radiomics models in the process of outlining the region of interest are manual-segmentation of the image and lack certain criteria.

Limitations of this study were that this was a retrospective and single-center study with a limited sample size. However, the result of the validation dataset from another ultrasound scanner, makes our radiomic model more convincing. Thus, future large multi-center studies are still warranted to assure the generalizability of our results. In addition, the diagnostic performance of the model in this study isn’t comparing with human readers. It is a challenge for a sonographer to directly determine whether a lymph node is metastatic without scanning it. As for the central lymph nodes, it’s more difficult due to impact of air, bone, or glandular tissues and the experience of the doctor. Futhermore, our study only using a single 2D ultrasound image, and did not incorporate other multimodal ultrasound images such as elastography, ultramicro flow imaging, contrast-enhanced ultrasound, etc.

## Conclusion

Our combined model is a noninvasive predictive tool that combined ultrasound radiomic and morphological features, which showed a better ability to predict CLNM preoperatively compared with ultrasound radiomic or morphology models, and can help clinicians to select more reasonable treatment modalities and avoid overtreatment.

### Electronic supplementary material

Below is the link to the electronic supplementary material.

Supplementary Material 1

Supplementary Material 2

Supplementary Material 3

Supplementary Material 4

Supplementary Material 5

## Data Availability

All data generated or analyzed during this study are included in its supplementary information files.

## Abbreviations

CLNM: Central lymph node metastasis
PTC: Papillary thyroid carcinoma
ROI: Region of interest
LASSO: Least absolute shrinkage selection operator
Rad-score: Radiomic score
AUC: Area under curve
OR: Odds ratio
95% CI: 95% confidence interval
CCND: Central compartment neck dissection
US: Ultrasonography
ICC: Intraclass correlation coefficient
GLCM: Gray level cooccurrence matrix
GLDM: Gray level dependence matrix
GLRLM: Gray level run length matrix
GLSZM: Gray level size zone matrix
NGTDM: Neighborhood gray tone difference matrix
DCA: Decision curve analysis
ROC: Receiver operating characteristic
CT: Computed tomography
MRI: Magnetic resonance imaging
