# Supplementary material for: Predicting central lymph node metastasis in patients with papillary thyroid carcinoma based on ultrasound radiomic and morphological features analysis
Source: BMC Med Imaging. 2023 Aug 24;23:111. doi: 10.1186/s12880-023-01085-4 (PMC10463837; doi:10.1186/s12880-023-01085-4)
Supplement: Supplementary file 1 — Supplementary Material 1 [file 12880_2023_1085_MOESM1_ESM.docx]

Table Legend

Table S1 Features extracted from 2D ultrasound images in the training set.

Table S2 Features extracted from 2D ultrasound images in the validation set.

Table S3 Clinical and ultrasound morphological features of the patients in the training set.

Table S4 Clinical and ultrasound morphological features of the patients in the validation set.
